# Mitochondrial translocation of cofilin is required for allyl isothiocyanate-mediated cell death via ROCK1/PTEN/PI3K signaling pathway

**DOI:** 10.1186/1478-811X-11-50

**Published:** 2013-07-29

**Authors:** Guo-bing Li, Qi Cheng, Lei Liu, Ting Zhou, Chang-yu Shan, Xiao-ye Hu, Jing Zhou, E-hu Liu, Ping Li, Ning Gao

**Affiliations:** 1 Department of Pharmacognosy, College of Pharmacy, 3rd Military Medical University, Chongqing 400038, China; 2 State Key Laboratory of Natural Medicines (China Pharmaceutical University), Nanjing 210009, China

**Keywords:** Allyl isothiocyanate, Apoptosis, Cofilin, ROCK1, PI3K, Leukemia

## Abstract

**Background:**

Cofilin is a member of the actin depolymerizing factor (ADF)/cofilin family, which regulates actin dynamics. Increasing evidence suggests that mitochondrial translocation of cofilin appears necessary for the regulation of apoptosis.

**Results:**

We report that allyl isothiocyanate (AITC) potently induces mitochondria injury and apoptosis. These events were accompanied by a loss of polymerized filamentous actin (F-actin) and increase in unpolymerized globular actin (G-actin). AITC also induces dephosphorylation of cofilin through activation of PP1 and PP2A. Only dephosphorylated cofilin binds to G-actin and translocates to mitochondria during AITC-mediated apoptosis. Mechanistic study revealed that interruption of ROCK1/PTEN/PI3K signaling pathway plays a critical role in AITC-mediated dephosphorylation and mitochondrial translocation of cofilin and apoptosis. Our *in vivo* study also showed that AITC-mediated inhibition of tumor growth of mouse leukemia xenograft model is in association with dephosphorylation of cofilin.

**Conclusions:**

These findings support a model in which induction of apoptosis by AITC stems primarily from activation of ROCK1 and PTEN, and inactivation of PI3K, leading in turn to activation of PP1 and PP2A, resulting in dephosphorylation of cofilin, which binds to G-actin and translocates to mitochondria, culminating in the dysfunction of mitochondria, release of cytochrome c and apoptosis.

## Background

Mitochondria are the major organelles involved in the biochemical execution of apoptosis [1]. Apoptotic proteins that target mitochondria may increase the permeability of the mitochondrial membrane and cause apoptotic effectors to leak out [2]. Cytochrome c is released from mitochondria into the cytoplasm and in turn activates/cleaves caspase-9 by forming a complex with apoptotic protease activating factor-1 (Apaf-1), leading to activating the effector caspase-3 [3]. Although the role of mitochondria in controlling downstream apoptotic events such as caspases activation is relatively well characterized, mechanisms by which upstream apoptotic signals are transduced to mitochondria remain largely elusive.

Actin cytoskeleton is involved in diverse cellular functions, including cell growth, motility, differentiation, as well as apoptosis [4]. Alteration of actin dynamics might be responsible for modulating apoptosis [5]. Recent data suggests that the activity of actin regulatory protein such as ADF/cofilin plays a critical role in the regulation of apoptosis in mammalian cells [6]. However, normal apoptosis in cofilin-1-deficient mouse fibroblasts argue against a general role of cofilin-1 in apoptosis progression [7]. Cofilin is a member of ADF/cofilin family, which regulates actin dynamics by increasing the rate of actin depolymerization and facilitating actin filament turnover [8]. It was recently shown that cofilin translocation to mitochondria appears necessary for the opening of the mitochondrial permeability transition pore and subsequent release of cytochrome c and the initiation of apoptosis [9]. Phosphorylation of cofilin at a single serine residue, Ser3, is a common mechanism regulating its activity [10]. Only dephosphorylated cofilin is involved in its mitochondrial translocation. In contrast, phosphorylation of cofilin at Ser 3 suppressed its mitochondrial translocation [9]. Phosphorylation/dephosphorylation of cofilin at Ser 3 is modulated by multiple regulatory kinases and phosphatases. Cofilin is phosphorylated at Ser3 by the LIM kinase LIMK and TES kinase TESK and is dephosphorylated by phosphatases such as slingshot, chronophin and PP1/PP2A [11-15]. Each of these phosphatases is regulated by different upstream signaling pathways such as PI3K [15].

Allyl isothiocyanate (AITC) is a naturally occurring compound that exhibits anticancer activity. Many commonly consumed cruciferous vegetables are rich sources of AITC [16]. Evidence supports that AITC exerts its antiproliferative effects through inducing cell cycle arrest and apoptosis in various cancer cell lines *in vitro* and in several tumor xenograft models *in vivo*[17]. For instance, AITC induces cell cycle arrest at G_2_/M phase and apoptosis in human bladder cancer cells and inhibits tumor growth of bladder cancer cell xenograft via JNK activation and Bcl-2 phosphorylation [18,19]. AITC induces G_2_/M cell cycle arrest and apoptosis in human breast cancer cells via ROS production and ERK signaling pathway [20]. AITC also induces apoptosis in human leukemia cells through cleavage of Bid and activation of JNK [21]. Recent studies suggest that a mitochondria-dependent pathway may play important roles in AITC-mediated apoptosis [18,20,22]. However, the molecular mechanism by which AITC regulates the mitochondrial pathway of apoptosis in human leukemia cells has not yet been explored.

In the present study, we provide evidence for the first time that AITC induces dephosphorylation of cofilin, which binds to G-actin and translocates to mitochondria, leading in turn to modifying the functional dynamics of actin cytoskeleton, resulting in the dysfunction of mitochondria, release of cytochrome c and apoptosis. Dephosphorylation of cofilin can be achieved with phosphatases PP1 and PP2A, which are regulated by ROCK1/PTEN/PI3K signaling pathway. Our *in vivo* results indicate that dephosphorylation of cofilin may contribute to AITC-mediated inhibitory effects on tumor growth of U937 xenograft mouse model. These findings provide a novel mechanistic basis for AITC as a leukemia treatment strategy.

## Results

### AITC potently induces mitochondrial injury and apoptosis in transformed and primary human leukemia cells

**Figure 1:**
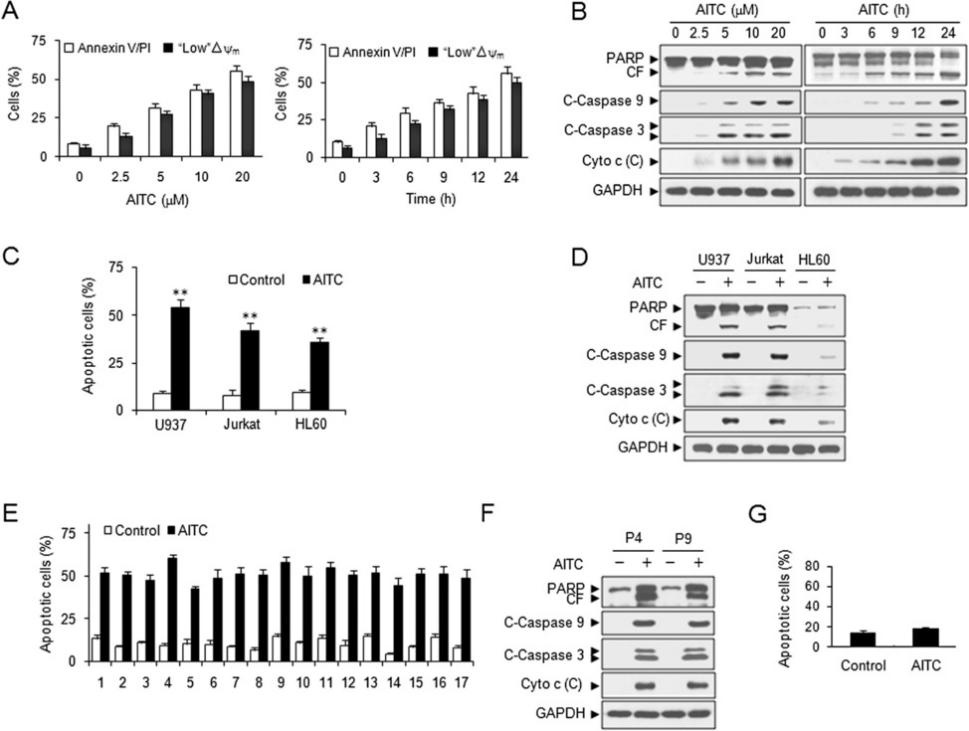
**AITC selectively induces apoptosis and mitochondrial injury in transformed and primary human leukemia cells.** U937 cells were treated without or with various concentrations of AITC for 24 h, or treated with 20 μM AITC for different time intervals as indicated. **(A)** Apoptosis and reduction in △ψ_m_ were determined using flow cytometry as described in Methods. ‘Low’ △ψ_m_ values are expressed as the percentage of cells exhibiting a diminished mitochondrial membrane potential. Error bars represent means ± SD for (n=3). **(B)** Total cellular extracts and cytosolic fractions **(C)** were prepared and subjected to Western blot analysis using antibodies against PARP, cleaved-caspase 3 (C-Caspase 3), cleaved-caspase 9 (C-Caspase 9) , cytochrome c (Cyto c) and GADPH to ensure equivalent loading. Two additional studies yielded equivalent results. **(C-D)** U937, Jurkat, and HL-60 cells were treated without or with 20 μM AITC for 24 h, flow cytometry was used to determine apoptosis, and Western blot assay was used to determine the expression of PARP, C-Caspase 3, C-Caspase 9, and Cyto c. Error bars represent means ± SD (n=3). ** *P*< 0.01 compared with control. **(E)** Primary leukemia blasts were isolated from the peripheral blood of 17 patients with AML as described in Methods. After exposure to 20 μM AITC for 24 h, apoptosis was determined using flow cytometry. Error bars represent means ± SD (n=3). **(F)** Whole cell lysates and cytosolic fractions **(C)** in blasts from 2 AML patients were obtained and subjected to Western blot analysis. **(G)** Normal CD34+ cells were exposed to 20 μM AITC for 24 h, after which apoptosis was determined using flow cytometry. Error bars represent means ± SD (n=3). *P* = 0.056.

Flow cytometry analysis revealed that exposure of cells to 5 μM AITC for 24 h resulted in a moderate increase in mitochondrial injury (loss of △ψ_m_) and apoptosis (Figure 1A). These events became apparent at 10 μM and very extensive at 20 μM concentrations. A time-course study of cells exposed to 20 μM AITC revealed a moderate increase in mitochondrial injury and apoptosis as early as 6 h after drug exposure. These events became apparent after 9 and 12 h of drug exposure and very extensive after 24 h of drug exposure (Figure 1A). Consistent with these findings, the same AITC concentrations and exposure intervals caused cleavage/activation of caspase-9 and caspase-3, and degradation of PARP. These events were also accompanied by release of cytochrome c into the cytosolic fraction (Figure 1B).

To determine whether these events were restricted to myeloid leukemia cells, parallel studies were performed in Jurkat and HL-60 leukemia cells. These cells exhibited apoptotic effects of AITC similar to those observed in U937 cells (Figure 1C). Also, Jurkat and HL-60 cells exhibited comparable degrees of caspase-9 and -3 activation and PARP degradation, and cytochrome c release (Figure 1D).

To determine whether AITC could also trigger apoptosis in primary human leukemia cells, primary leukemia cells isolated from 17 AML patients were treated without or with 20 μM AITC for 24 h, after which apoptosis was determined by Annexin V/PI analysis. Exposure of these AML blasts to AITC resulted in marked increase in apoptosis (Figure 1E). Consistent with these findings, treatment of leukemia blasts from 2 AML patients with AITC also resulted in cleavage/activation of caspase-9 and -3, degradation of PARP, and release of cytochrome c (Figure 1F). In contrast, AITC exerted little toxicity toward normal CD34+ bone marrow cells (Figure 1G). Taken together, these findings suggest that AITC selectively induces mitochondrial injury and apoptosis in transformed and primary human leukemia cells but not in normal hematopoietic cells.

### Alteration of G/F-actin ratio and actin dynamics in response to AITC

**Figure 2:**
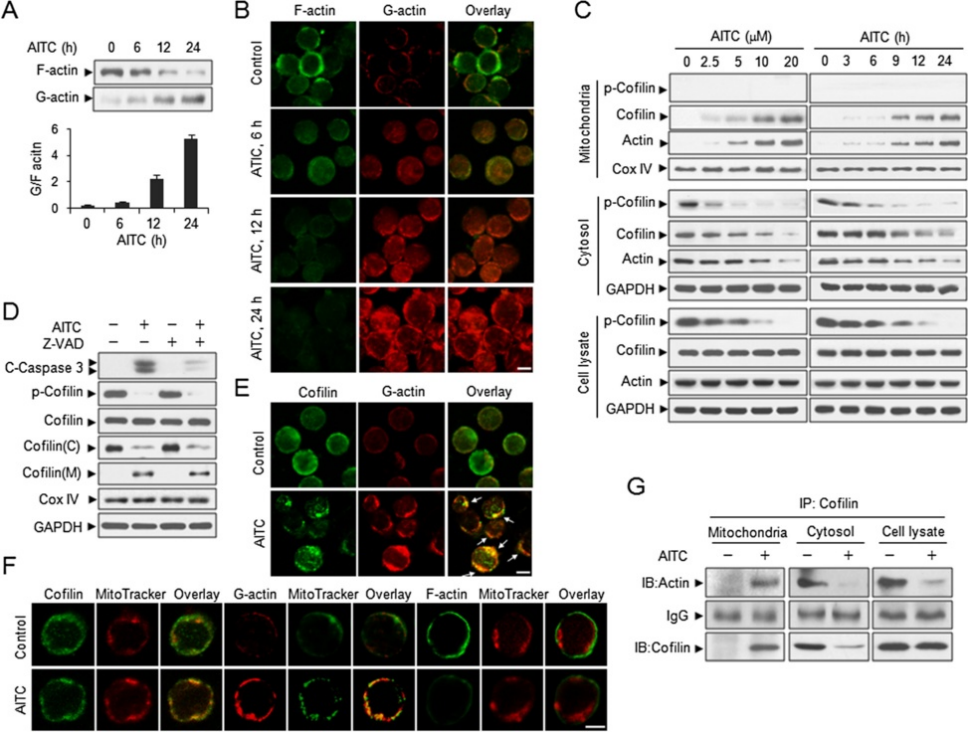
**AITC induces shift in G/F-actin, translocation/localization in mitochondria and dephosphorylation of cofilin. (A)** U937 cells were treated with 20 μM AITC for different time intervals as indicated, the levels of F-actin and G-actin were measured using the G-actin/F-actin In Vivo Assay Kit as described in Methods. Densitometric analysis of the blots were performed by using Quantity One software (Bio-Rad laboratories, Munchen, Germany) and G/F-actin ratio was calculated. **(B)** Levels of F-actin (green) and G-actin (red) in cells treated with AITC were imaged by confocal microscopy. Scale bar represents 5 μm. **(C)** U937 cells were treated without or with various concentrations of AITC for 24 h, or treated with 20 μM AITC for different time intervals as indicated. Cell lysates, mitochondrial and cytosolic fractions were prepared and subjected to Western blot analysis using antibodies against phospho-cofilin (p-cofilin), cofilin and actin. Blots were subsequently stripped and reprobed with antibody against GADPH and COX IV (mitochondrial fraction) to ensure equivalent loading. **(D)** U937 cells were pretreated with the caspase inhibitor Z-VAD-FMK (20 μM) for 2 h, followed by treatment with 20 μM AITC for 24 h. Cell lysates, mitochondrial and cytosolic fractions were prepared and subjected to Western blot analysis. **(E-F)** Cells were untreated (control) or treated with 20 μM AITC for 12 h, cofilin was labeled by an anti-cofilin antibody followed by Alexa 488-conjugated goat anti-mouse antibody (green); fluorescent deoxyribonuclease I conjugates (red) for staining G-actin; Fluorescent phallotoxins for staining F-actin; mitochondria were stained by Mitotracker Red or Green as described in Methods. Fluorescence images were collected at similar focal planes by confocal microscopy. Scale bar represents 5 μm. **(G)** Cell lysates, cytosolic and mitochondrial fractions of control and AITC-treated cells were prepared and subjected to immunoprecipitation using an anti-cofilin antibody, followed by immunoblotting analysis.

G/F-actin ratio is an indicator of the extent of actin dynamics and might be responsible for regulating apoptosis [5]. To understand the mechanism of AITC-mediated apoptosis through affecting actin dynamics, we separated actin into G and F fractions and evaluated their relative content. Exposure of cells to AITC resulted in decrease in the polymerized F-actin and increase in the unpolymerized G-actin (Figure 2A). Consistent with these findings, confocal microscopy showed that treating with AITC for different time intervals resulted in decrease in levels of F-actin and increase in levels of G-actin (Figure 2B). Such findings suggest that AITC induces actin dynamics through filamentous actin depolymerization.

### Dephosphorylation of cofilin during AITC-mediated apoptosis is essential for its mitochondrial translocation

Since actin dynamics is regulated by actin depolymerization factor cofilin [23], next we examined the levels of cofilin and actin either in mitochondrial and cytosolic fractions or total cellular extracts using Western blot analysis. Treating cells with AITC resulted in marked increase in levels of cofilin and actin in mitochondrial fraction and decrease in levels of cofilin and actin in cytosolic fraction in dose- and time-dependent manners (Figure 2C). In contrast, AITC treatment had little or no effect on the expression of cofilin and actin in total cellular extract. On the other hand, translocation of cofilin from cytosol to mitochondria after AITC exposure were also observed in Jurkat and HL-60 cells, but not in normal CD34+ bone marrow cells (Additional file 1: Figure S1 A-B). These findings indicate that mitochondrial translocation of cofilin may occur through increased of actin with mitochondria during AITC-mediated apoptosis.

We then investigated whether the phosphorylation status of cofilin can influence its ability to translocate to mitochondria and induce apoptosis. Phosphorylated cofilin was identified in the cytosolic fraction and total cellular extract of control cells. When cells were treated with AITC, cofilin was dephosphorylated in both the cytosolic fraction and total cellular extract in dose- and time-dependent manners (Figure 2C). However, phosphorylated cofilin was not identified in mitochondrial fraction of control and AITC-treated cells.

We also investigated the effects of caspase inhibition by Z-VAD-FMK on dephosphorylation and mitochondrial translocation of cofilin mediated by AITC. Dephosphorylation and mitochondrial translocation of cofilin caused by AITC were not blocked by the caspase inhibitor Z-VAD-FMK (Figure 2D), suggesting that dephosphorylation and mitochondrial translocation of cofilin is a common phenomenon that occurs before caspase activation.

Since dephosphorylated cofilin binds actin with high affinity, whereas the phosphorylated forms cannot interact [24]. We then investigated whether the association and co-localization of cofilin and G-actin could be involved in AITC-mediated apoptosis using immunofluorescence microscopy. The results showed that cofilin and G-actin were co-localized in cells treated with AITC (Figure 2E). Because the above results showed that treating cells with AITC resulted in marked increase in levels of cofilin and actin in mitochondrial fraction, we then investigate sub-cellular localization of cofilin during AITC induces apoptosis by immunofluorescence microscopy with the mitochondrion selective probe Mitotracker Red CMXRos and cofilin. In control cells, total cofilin levels in mitochondria were not observed. However, when cells were treated with AITC, cofilin signal was localized in the mitochondria (Figure 2F). We also investigated whether actin was localized in mitochondria by immunofluorescence microscopy with the mitochondrion selective probe Mitotracker Green FM/MitoTracker Red CMXRos and G-actin/F-actin. Surprisingly, localization of G-actin but not F-actin in mitochondria was observed after AITC exposure (Figure 2F).

We further investigated whether AITC affects the association between cofilin and actin in cell lysates, cytosolic and mitochondrial fractions. Immunoprecipitation of cell lysates and cytosolic fraction with an anti-cofilin antibody revealed that actin co-precipitated with cofilin in cell lysates and cytosolic fraction of control cells. However, this actin-cofilin complex was lost after AITC exposure (Figure 2G). Interestingly, the actin-cofilin complex was observed in mitochondrial fraction after AITC exposure (Figure 2G). Taken together, these findings indicate that AITC dephosphorylates cofilin, which binds with G-actin, leading to translocation to mitochondria, culminating in cytochrome c release and apoptosis.

### Dephosphorylation of cofilin by PP1 and PP2A is required for AITC-induced apoptosis

**Figure 3:**
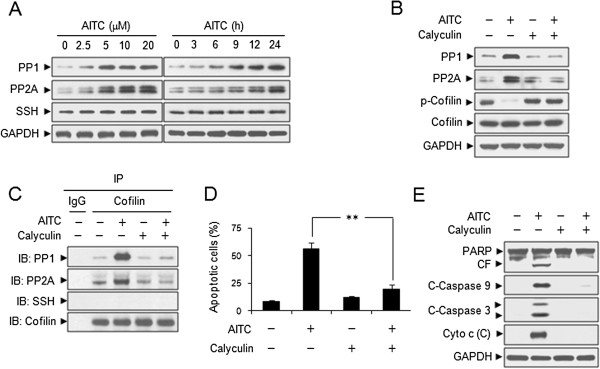
**AITC induces dephosphorylation of cofilin by PP1 and PP2A. (A)** U937 cells were treated without or with various concentrations of AITC for 24 h, or treated with 20 μM AITC for different time intervals as indicated. Total cellular extracts were prepared and subjected to Western blot analysis using antibodies against PP1, PP2A and slingshot (SSH). Cells were pretreated with 50 nM calyculin, a PP1/PP2A inhibitor, for 2 h, followed by treating with 20 μM AITC for 24 h. **(B)** Total cellular extracts were prepared and subjected to Western blot analysis using antibodies against PP1, PP2A, p-cofilin, and cofilin. **(C)** Cell lysates were prepared and subjected to immunoprecipitation using anti-cofilin antibody. The associated PP1, PP2A, SSH, and cofilin were determined using immunoblotting. **(D)** Cell apoptosis was determined by Annexin V/PI staining and flow cytometry. Error bars represent means ± SD (n=3). ** *P* < 0.01. **(E)** Total cellular extracts and cytosolic fractions were prepared and subjected to Western blot analysis using antibodies as indicated.

Since cofilin is activated/dephosphorylated by phosphoatase enzymes such as PPases (PP1 and PP2A) and slingshot [24], we examined whether AITC induces the expression of these phosphatases. Treating cells with AITC resulted in marked increase in levels of PP1 and PP2A in dose- and time-dependent manners, whereas the expression of slingshot was not altered by AITC (Figure 3A). These events also occurred in Jurkat and HL-60 cells, but not in normal CD34+ bone marrow cells (Additional file 1: Figure S1 A-B). To investigate the role of PP1 and PP2A in AITC mediated dephosphorylation of cofilin, a PP1/PP2A inhibitor, calyculin, was employed. Co-treatment of cells with calyculin significantly abrogated AITC-mediated dephosphorylation of cofilin and expression of PP1 and PP2A (Figure 3B). To further confirm that PP1 and PP2A are responsible for dephosphorylation of cofilin mediated by AITC, we used immunoprecipitaion assays with an anti-cofilin antibody. Immunoprecipitation of cofilin pulled down PP1 and PP2A after AITC exposure, whereas slingshot was not detected (Figure 3C). Inhibition of PP1 and PP2A by calyculin also blocked the PP1/PP2A–cofilin interactions (Figure 3C). We further studied whether inhibition of PP1 and PP2A is sufficient to prevent cells from the effects of AITC on apoptosis. Co-treatment of cells with calyculin markedly abrogated AITC-induced apoptosis, caspases activation, PARP degradation and cytochrome c release (Figure 3D and E). Together, our data reveal that dephosphorylation of cofilin is caused mainly by induction of PP1 and PP2A phosphatase activities, which are responsible for AITC-induced apoptosis in leukemia cells.

### Inhibition of PI3K activity is responsible for AITC-induced PP1 and PP2A activation and cofilin dephosphorylation and translocation to mitochondria

**Figure 4:**
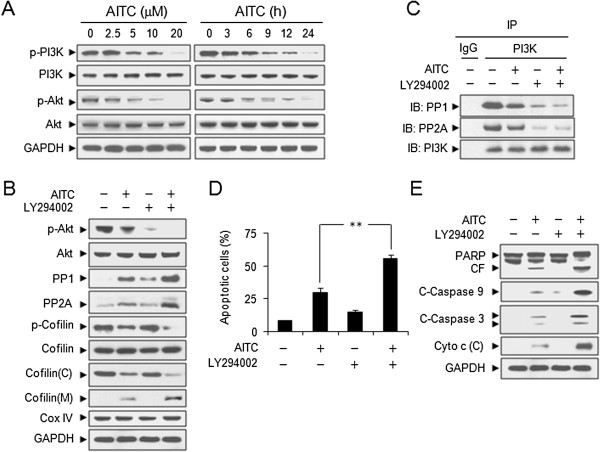
**Inhibition of PI3K activity contributes to AITC-induced activation of PP1/PP2A and mitochondrial translocation of cofilin. (A)** U937 cells were treated without or with various concentrations of AITC for 24 h, or treated with 20 μM AITC for different time intervals as indicated. Total cellular extracts were prepared and subjected to Western blot analysis using antibodies against phospho-PI3K (p-PI3K), PI3K, phospho-Akt [p-Akt (Ser473)], and Akt. Cells were pretreated with 20 μM LY294002, a specific PI3K inhibitor, for 2 h, followed by treating with 5 μM AITC for 24 h. **(B)** Total cellular extracts, cytosolic and mitochondrial fractions were prepared and subjected to Western blot analysis. **(C)** Cell lysates were prepared and subjected to immunoprecipitation using anti-PI3K antibody. The associated PP1 and PP2A were determined using immunoblotting. **(D)** Apoptosis was determined by flow cytometry. Error bars represent means ± SD (n=3). **P < 0.01. **(E)** Total cellular extracts and cytosolic fractions were prepared and subjected to Western blot analysis using antibodies as indicated.

It has recently shown that phosphatase PP2A activity is regulated negatively by PI3K signaling [25]. To investigate whether PI3K plays a role in AITC-induced cofilin dephosphorylation and mitochondrial translocation and PP1 and PP2A activation, we first examined the effects of AITC on PI3K activity and its substrate, Akt phosphorylation. Exposure of cells to AITC resulted in decrease in levels of phospho-PI3K and phospho-Akt (Ser473) in dose- and time-dependent manners (Figure 4A). These events also occurred in Jurkat and HL-60 cells, but not in normal CD34+ bone marrow cells (Additional file 1: Figure S1 A-B).

We used specific PI3K inhibitor LY294002 to examine the effects of inhibition of PI3K activity on dephosphorylation and mitochondrial translocation of cofilin and activation of PP1 and PP2A induced by AITC. Co-administration of AITC and LY294002 resulted in the virtual abrogation of Akt activation. Pretreatment with LY294002 also significantly enhanced AITC-mediated dephosphorylation and mitochondrial translocation of cofilin and activation of PP1 and PP2A (Figure 4B). To further elucidate the mechanism of PP1 and PP2A regulation by PI3K, we examined the binding of PP1 or PP2A and PI3K using immunoprecipitation analysis. Our results revealed the significant decrease in the association between PP1 or PP2A and PI3K after AITC exposure (Figure 4C). Pretreatment with LY294002 also enhanced the decrease in the association between PP1 or PP2A and PI3K mediated by AITC (Figure 4C). We further studied whether inhibition of PI3K activity by LY294002 may enhance AITC-induced apoptosis. Co-treatment with LY294002 significantly promoted AITC-induced apoptosis, caspases activation, PARP degradation and cytochrome c release (Figure 4D and E). Together, these findings suggest that inactivation of PI3K plays a critical role in AITC-induced cofilin dephosphorylation and mitochondrial translocation and apoptosis through activation of PP1 and PP2A.

### ROCK1/PTEN signaling pathway is involved in AITC-mediated PI3K inactivation, cofilin dephosphorylation and mitochondrial translocation, and apoptosis

**Figure 5:**
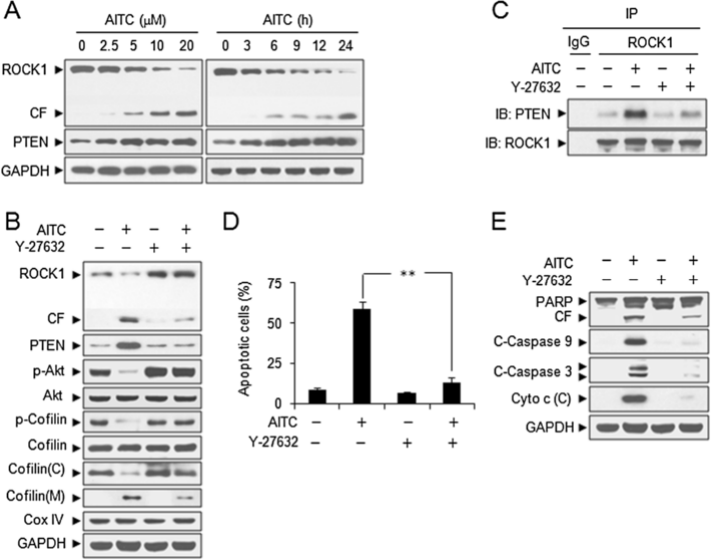
**ROCK1/PTEN/PI3K signaling pathway regulates AITC-mediated dephosphorylation and mitochondrial translocation of cofilin and apoptosis. (A)** U937 cells were treated without or with various concentrations of AITC for 24 h, or treated with 20 μM AITC for different time intervals as indicated. Total cellular extracts were prepared and subjected to Western blot analysis using antibodies against ROCK1 and PTEN. CF represents cleavage fragment. Cells were pretreated with 20 μM Y27632, a ROCK1 inhibitor, for 2 h, followed by treating with 20 μM AITC for 24 h. **(B)** Total cellular extracts, cytosolic and mitochondrial fractions were prepared and subjected to Western blot analysis. **(C)** Cell lysates were prepared and subjected to immunoprecipitation using anti-ROCK1 antibody. The associated PTEN was determined using immunoblotting. **(D)** Apoptosis was determined by Annexin V/PI staining and flow cytometry. Error bars represent means ± SD (n=3). **P < 0.01. **(E)** Total cellular extracts and cytosolic fractions were prepared and subjected to Western blot analysis using antibodies as indicated.

It has been shown that PTEN is a PI3K upstream negative regulator and is regulated by ROCK1 [26]. Next we examined the effects of AITC on the expression of ROCK1 and PTEN. Treating cells with AITC resulted in marked decrease in levels of ROCK1 and increase in cleavage of ROCK1, and increase in levels of PTEN in dose- and time-dependent manners (Figure 5A). These events also occurred in Jurkat and HL-60 cells, but not in normal CD34+ bone marrow cells (Additional file 1: Figure S1 A-B). To determine the functional significance of ROCK1 in regulation of PTEN activity and down-stream molecules during AITC-induced apoptosis, we then examined the effects of inhibition of ROCK1 by Y27632 on the expression of ROCK1, PTEN, phospho-Akt, and phospho-cofilin. Co-administration of Y27632 significantly blocked AITC-mediated ROCK1 activation, PTEN activity, Akt inactivation and cofilin dephosphorylation. Furthermore, Co-administration of Y27632 significantly blocked AITC-mediated cofilin translocation to mitochondria (Figure 5B). To further elucidate the mechanism of PTEN regulation by ROCK1, we examined the binding of PTEN and ROCK1 in response to AITC treatment in leukemia cells. Our results revealed the significant increase in the association between PTEN and ROCK1 after AITC exposure (Figure 5C). Pretreatment with Y27632 markedly abrogated the association between PTEN and ROCK1 mediated by AITC (Figure 5C). We further studied whether inhibition of ROCK1 is sufficient to prevent cells from the effects of AITC on apoptosis. Co-treatment of cells with Y27632 markedly abrogated AITC-induced apoptosis, activation of caspases-3 and -9, degradation of PARP and cytochrome c release (Figure 5D and E). Taken together, these results demonstrate that ROCK1 plays a significant role in regulating the activation of PTEN in response to AITC treatment in leukemia cells, which probably contributes to dephosphorylation and mitochondrial translocation of cofilin and induction of apoptosis.

### AITC inhibits tumor growth of U937 xenograft model accompanied by striking induction of apoptosis and dephosphorylation of cofilin

**Figure 6:**
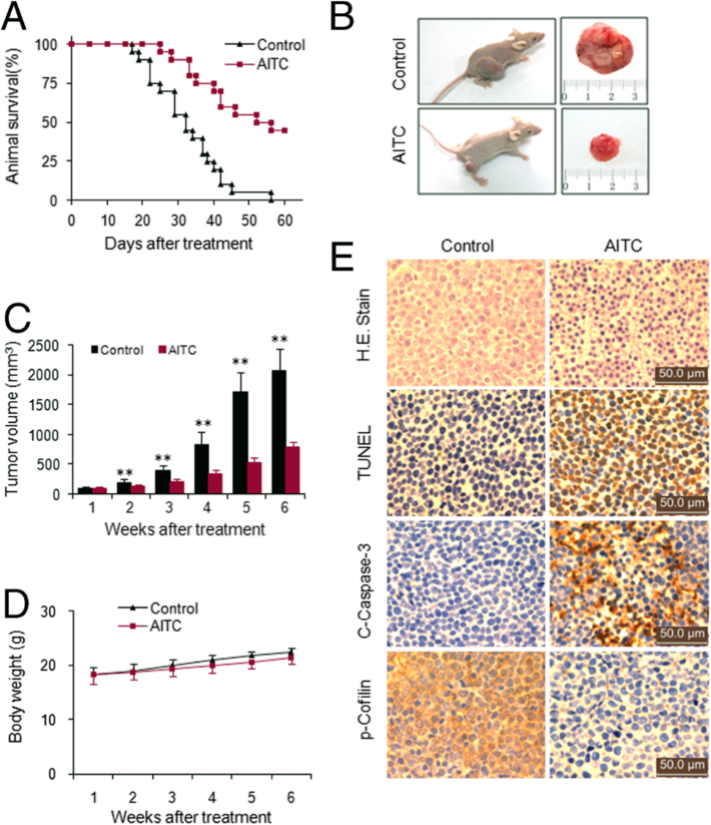
**AITC inhibits tumor growth and induces apoptosis in U937 xenografts animal model.** 40 nude mice were inoculated with U937 cells (2 × 10^6^ cells/mouse, s.c.) and randomly divided into two groups (20/group) for treatment with AITC (50 mg/kg, i.p., daily, five times per week) or with vehicle control solvent as described in Methods. Tumor growth was measured once a week, and tumor volume (V) was calculated as V = (L × W^2^) × 0.5, where L is the length and W is the width of a xenograft. **(A)** Comparison of animal survival of AITC-treated group and vehicle-treated group (*P* < 0.01). **(B)** Gross appearance of two mice with solid tumor of vehicle control group and AITC-treatment group. **(C)** Average tumor volume in vehicle control mice and mice treated with 50 mg/kg AITC. Error bars represent means ± SD. *P < 0.05 or **P < 0.01 compared with vehicle control. **(D)** Body weight changes of mice during the six weeks of study. Statistical analysis of body weight changes showed no significant differences between AITC treatment and vehicle control groups. **(E)** After treatment with AITC (50 mg/kg), tumor tissues were sectioned and subjected to H&E staining, TUNEL assay, and immunohistochemistry for evaluating histological morphology, apoptosis and expression of C-Caspase-3 and p-cofilin.

The ability of AITC in killing both transformed and primary human leukemia cells *in vitro* led us to evaluate its antileukemic activity *in vivo*. Nude mice were inoculated subcutaneously with U937 cells, after which mice were received injections with vehicle or AITC (50 mg/kg, i.p.) for 6 weeks starting five days after the injection of U937 human leukemia cells. Treatment with AITC resulted in a significant increase in the median survival compared with untreated controls (52 vs 32 days, *P*< 0.01) (Figure 6A). Only the subcutaneous inoculation of U937 cells into nude mice resulted in a tumor formation at the site of injection in all mice (Figure 6B). Treatment with AITC resulted in a modest but significant suppression of tumor growth 2 weeks following drug exposure (**P < 0.01 vs vehicle control). These events became more apparent 3 weeks and 4 weeks and very extensive 5 weeks and 6 weeks after drug exposure (**P < 0.01 vs vehicle control) (Figure 6B and C). However, no significant changes in weight or other signs of potential toxicity such as agitation, impaired movement and posture, indigestion or diarrhea, and areas of redness or swelling were observed during the treatment with AITC (Figure 6D).

We then examined the morphological changes and induction of apoptosis in tumor section of U937 xenografts using H&E staining, TUNEL and immunohistochemistry analysis. The sections of U937 xenografts from mice treated with AITC exhibited a reduced number of cancer cells, with signs of necrosis with infiltration of inflammatory cells and fibrosis (Figure 6E, top panels). Exposure to AITC resulted in a striking induction of apoptosis in tumor cells, with signs of numerous dark brown colored apoptotic cells (Figure 6E, second panels). In addition, treatment with AITC caused a rapid increase in immunoreactivity for cleaved caspase-3, indicative of apoptosis (Figure 6E, third pannels). Finally, we applied immunohistochemistry analysis to evaluate whether AITC induces dephosphorylation of cofilin *in vivo*. Treatment with AITC resulted in markedly decrease in expression of phospho-cofilin in tissue sections of tumors (Figure 6E, bottom pannels). However, AITC had no effect on total cofilin levels in tissue sections of tumors (data not shown). Such findings suggest that AITC-mediated antileukemic activity *in vivo* is associated with dephosphorylation of cofilin.

## Discussion

Actin cytoskeleton plays an important role in diverse cellular functions, including cell growth and apoptosis [4]. Recent research has highlighted a role of actin cytoskeleton in the initiation or inhibition of apoptotic processes. Apoptosis can be triggered by jasplakinolide in Jurkat T cells through the stabilization of F-actin and these events are accompanied by an increase in caspase-3 activation [27]. Apoptosis can also be triggered by the destabilization of F-actin structures. For example, treatment with cytochalasin D resulted in rapid cytochrome c release from mitochondria, caspase activation and apoptosis in murine cell lines [28]. Our present results indicate that AITC-induced apoptosis is probably resulted from actin cytoskeletal rearrangement, because both immunofluorescence staining and immunoblotting showed a dramatic decrease of polymerized F-actin and a concomitant increase of unpolymerized G-actin in AITC-treated cells. Since a dynamic switch between monomeric G-actin and F-actin may be involved in apoptotic processes, the suppression of actin polymerization by AITC might contribute to its effects on apoptosis.

Cofilin is a member of ADF/cofilin family, which regulates actin dynamics by increasing the rate of actin depolymerization [8]. It has been shown that mitochondrial translocation of cofilin was found to induce apoptosis in neutrophils [28]. Cofilin was found to translocate to mitochondria after staurosporine-induced apoptosis in a neuroblastoma cell [9]. These results suggest that the actin-binding activity of cofilin is crucial for its apoptosis-inducing activity. As dephosphorylated cofilin binds to actin and translocates to mitochondria, actin cytoskeletal changes may affect mitochondria function, resulting in the release of cytochrome c and apoptosis [9]. Rehklau, et al reported that translocation of cofilin to mitochondria is highly dependent on actin that also translocates to mitochondria during apoptosis [7]. These results suggest that the mitochondrial translocation of actin during apoptosis is essential for the mitochondrial association with cofilin. However, the molecular mechanism by which actin translocates to mitochondria and binds with cofilin remains elusive. Consistent with these studies, our results indicate that stimulation of apoptosis with AITC resulted in mitochondrial translocation of both cofilin and actin. Interestingly, only G-actin but not F-actin was translocated to mitochondria during AITC-induced apoptosis through the association with cofilin, based on the following findings: (i) The co-localization of cofilin and G-actin was observed in cells treated with AITC; (ii) Cofilin was co-localized with the mitochondrial marker Mitotracker Red CMXRos, and G-actin was aslo co-localized with the mitochondrial marker Mitotracker Green FM; (iii) The association of cofilin and G-actin was observed in mitochondrial fraction during AITC-induced apoptosis. To the best of our knowledge, this is the first report that the association and mitochondrial translocation of cofilin and G-actin are required for AITC-mediated cell death.

It has been shown that only dephosphorylated cofilin translocates to mitochondria during apoptosis, whereas phosphorylation of cofilin suppressed mitochondrial translocation [7,9]. Dephosphorylation of cofilin occurs in response to many stimuli that activate cells in processes requiring changes in actin dynamics [29]. As dephosphorylation of cofilin can be regulated by phosphatases such as slingshot and PPases including PP1 and PP2A [12,13], activation of these phosphatases may be required for translocation of cofilin to mitochondria during apoptosis. Our experiments showed that only dephosphorylated cofilin accumulated in mitochondria after induction of apoptosis by AITC. We also found that PP1 and PP2A, but not slingshot, were involved in dephosphorylation of cofilin induced by AITC. Inhibition of PP1 and PP2A with calyculin, a phosphatase inhibitor, which prevented AITC-induced dephosphorylation of cofilin and protected cells from AITC-mediated cell death. PP1 and PP2A are two major serine/threonine phosphatases, which dephosphorylate a large number of targets including components of the actin cytoskeleton [30]. Their recruitment for cofilin dephosphorylation may be crucial for the actin-binding activity and mitochondiral traslocation of cofilin. This in turn indicates the necessity for actin cytoskeletal changes, resulting in the dysfunction of mitochondria, release of cytochrome c and apoptosis.

Numerous studies showed that the activity of PPases are regulated by different upstream signaling pathways [11]. For example, PI3K negatively regulates PP2A activity that has significant consequences on receptor function with broad implications in cellular signaling [25]. Our findings demonstrate that inactivation of PI3K may contributed AITC-mediated activation of PP1 and PP2A and dephosphorylation and mitochondrial translocation of cofilin, based on the following evidence: (i) AITC inhibited PI3K activity and Akt activation in dose- and time-dependent manners; (ii) Pretreatment with PI3K inhibitor LY294002 not only enhanced AITC-inhibited Akt activation but also enhanced AITC-induced activation of PP1 and PP2A and dephosphorylation and mitochondrial translocation of cofilin; (iii) Pretreatment with LY294002 enhanced AITC-mediated cell death. It has been well documented that PTEN is a negative regulator of PI3K/Akt pathway, which has important roles in a diverse range of biological processes, including cell death and survival [31]. Recent evidence revealed that PTEN is a newly identified ROCK substrate, which is involved in the regulation of cell death and survival [26]. A number of evidence indicates that RhoA/ROCK1 activation enhances PTEN activity and suppresses Akt activation. For instance, PTEN mediates the reduction of Akt phosphorylation induced by ROCK activation in HEK cells [32]. RhoA and its effector kinase ROCK inhibit PI3K activity by enhancing the activity of PTEN, which negatively regulates PI3K/Akt signaling pathway [33]. Consistence with these reports, our findings suggest that activation of ROCK1 and PTEN and inactivation of PI3K could contribute to AITC-induced activation of PP1 and PP2A and dephosphorylation and mitochondrial translocation of cofilin, leading to apoptosis. Specifically, AITC exposure resulted in activation of ROCK1 and PTEN, and inactivation of PI3K/Akt. Inhibition of ROCK1 activity by Y27632 attenuated AITC-mediated apoptosis through preventing PTEN activity, Akt inactivation, cofilin dephosphorylation and mitochondrial translocation.

## Conclusions

**Figure 7:**
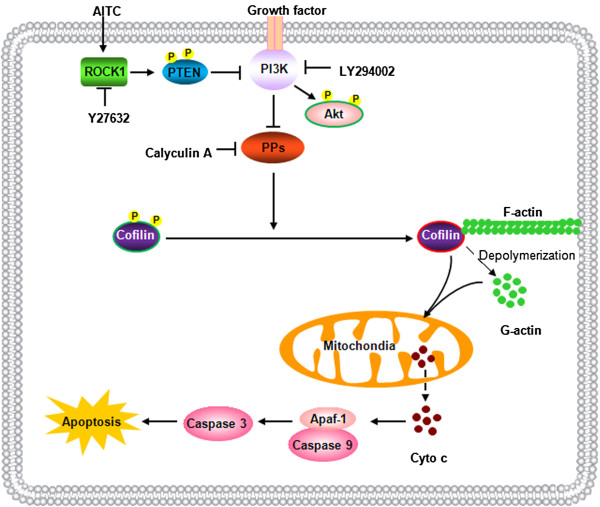
**A proposed model for AITC-mediated mitochondrial injury and apoptosis.** AITC-induced cell death in which ROCK1 activation represents the primary insult, leading in turn to PTEN activation and PI3K inactivation, resulting in PP1 and PP2A activation, and dephosphorylation of cofilin, which binds to G-actin and translocates to mitochondria, and culminating in cytochrome c release, caspases activation and apoptosis.

Our data indicate that AITC effectively induces apoptosis and mitochondrial injury in transformed and primary human leukemia cells and U937 tumor xenografts. Collectively, these findings suggest a hierarchy of events in AITC-induced apoptosis in which ROCK1 activation represents the primary insult, leading in turn to PTEN activation and PI3K inactivation, resulting in PP1 and PP2A activation, forming dephosphorylated cofilin, which binds to G-actin and translocates to mitochondria, culminating in dysfunction of mitochondria, release of cytochrome c and apoptosis (Figure 7). Further efforts to understand the mechanism(s) by which AITC induces apoptosis in human leukemia cells *in vitro* and *in vivo* could improve treatment outcomes for hematologic malignancies.

## Methods

### Chemicals and antibodies

AITC was purchased from Sigma (St Louis, MO). Y-27632 was purchased from Santa Cruz Biotechnology (Santa Cruz, CA). LY294002 and Z-VAD-FMK purchased from EMD Biosciences (La Jolla, CA). Antibodies against Akt, cytochrome c, cofilin, actin, PP1, PTEN and GAPDH were from Santa Cruz Biotechnology (Santa Cruz, CA); cleaved caspase-3, cleaved caspase-9, phospho-Akt (Ser473), phosphor-Cofilin (Ser3), PI3K, phospho-PI3K and Cox IV were from Cell Signaling Technology (Beverly, MA); ROCK1 and SSH were from Abcam (Burlingame, CA); PP2A was from BD Bioscience (San Jose, CA); PARP was from Biomol (Plymouth Meeting, PA).

### Cell culture

U937, HL-60, and Jurkat cells were obtained from the American Type Culture Collection (Manassas, VA) and cultured in RPMI 1640 medium supplemented with 10% fetal bovine serum (FBS) and antibiotics. Cells were cultured at 37°C in a humidified atmosphere and 5% CO_2_ in air.

After approval by the Southwest Hospital Institutional Review Board (Chongqing, China), peripheral-blood samples were obtained from 17 patients with newly diagnosed or recurrent acute myeloid leukemia (AML) after acquiring informed consent. AML blasts were isolated by density gradient centrifugation over Histopaque-1077 (Sigma-Aldrich Co., St Louis, MO) at 600 g for 15 minutes. Isolated mononuclear cells were counted, re-suspended in RPMI 1640 medium at 8×10^5^/mL for treatment. CD34+ cells from bone marrow mononuclear cells of healthy donors were isolated using the MACS cell isolation kit (Miltenyi Biotec, BG, German) according to the manufacturer’s instructions. After washing and enumerating as described for mononuclear cells, cells were suspended at 8×10^5^/mL prior to treatment.

### Apoptosis and mitochondrial transmembrane potential assay

Cells were harvested and apoptosis was analyzed by flow cytometry using the Annexin V/PI staining kit (PharMingen, San Diego, CA) according to the manufacturer’s instructions. Briefly, 1×10^6^ cells were washed twice with phosphate-buffered saline (PBS), and stained with 5 μl of Annexin V-FITC and 10 μl of PI (5 μg/mL) in 1× binding buffer (10 mM HEPES, pH 7.4, 140 mM NaOH, 2.5 mM CaCl2) for 15 min at room temperature in the dark. The apoptotic cells were determined using a Becton-Dickinson FACScan cytoflurometer (Mansfield, MA, USA). Both early apoptotic (Annexin V-positive, PI-negative) and late (Annexin V-positive and PI-positive) apoptotic cells were included in cell death determinations.

A diminished mitochondrial membrane potential (△ψm) was monitored using DiOC_6_. For each condition, 4 × 10^5^ cells were incubated in 1 mL 40 nM DiOC_6_ at 37°C in for 15 minutes and subsequently analyzed using a Becton Dickinson FACScan cytofluorometer with excitation and emission settings of 488 and 525 nm, respectively.

### Preparation of mitochondrial and cytosolic fractions

Mitochondrial and cytosolic fractions were obtained as previously described [34]. Briefly, cell pellets were washed twice with PBS and resuspended in 5 × buffer A (20 mM HEPES, 10 mM KCl, 1.5 mM MgCl_2_, 1 mM EDTA, 1 mM EGTA, 1 mM Na_3_VO_4_, 2 mM leupeptin, 1 mM PMSF, 1mM DTT, 2 mM pepstatin, and 250 mM sucrose). Cells were homogenized by passing them through a 22-gauge needle 25 times. The homogenate was centrifuged in three sequential steps: 1000 g, 10,000 g, and 100,000 g. The 10,000 g pellet was considered the “mitochondrial” fraction, and the 100,000 g supernatant the “cytosolic” fraction. These fractions were subjected to Western blot and immunoprecipitation analyses.

### G-actin/F-actin assay

G-actin/F-actin assay were performed by using G-actin / F-actin In Vivo Assay Kit (Cytoskeleton, Denver, CO) according to the manufacturer’s instructions. Briefly, cells were lysed with LAS2 buffer (containing lysis, F-actin stabilization buffer, ATP stock solution and protease inhibitor cocktail stock solution) at 37°C for 1 h. Unbroken cells were removed by centrifugation at 2,000 rpm for 5 min. Cell lysates were then centrifuged at 100,000 g for 1 h, finally F-actin in the pellet and G-actin in the supernatant. Samples were mix with 5×SDS sample buffer and then analyzed by western blot with antibody against actin.

### Western blot and immunoprecipitation analysis

Cells were lysed in 1× NuPAGE LDS sample buffer supplemented with 50 mM dithiothreitol. The proteins were separated by SDS-PAGE, transferred to nitrocellulose membranes, and processed for immunoblotting as previously described [35]. For immunoprecipitation analysis, Cells were lysed in 1% NP-40 buffer (50 mM Tris (pH 7.4), 150 mM NaCl, 1% Nonidet P-40, 10% glycerol, 1 mM PMSF, 10 μg/mL aprotinin, 10 μg/mL leupeptin, 1 mM Na_3_VO_4_). Equal quantities of proteins were incubated with primary antibodies at 4°C on a rocking platform. Immune complexes were collected with protein G agarose beads (Pierce Biotechnology, Rockford, IL) followed by several washes in lysis buffer, samples were boiled and then subjected to SDS-PAGE/Western blot.

### Immunofluorescence

Cells were collected by centrifugation, resuspend gently in pre-warmed (37°C) staining solution containing 200 nM MitoTracker Red CMXRos (Molecular Probes, Eugene, OR) for 1 h at 37°C, and washed twice with RPMI 1640 medium. After fixed with 3.7% of methanol-free formaldehyde for 15 min, and permeabilized with 0.1% Triton X-100 for 10 min. Slides were blocked with 1% BSA in PBS for 30 min, then incubated with anti-cofilin primary antibody at 4°C overnight, followed by the secondary Alexa 488-conjugated goat anti-mouse antibody (Molecular Probes, Eugene, OR) for 1 h at room temperature. Cells were incubated with 50 nM MitoTracker Green FM (Molecular Probes, Eugene, OR) after fixation because it can not retained well after fixation. Fluorescent staining of globular and filamentous actin was performed using Fluorescent Deoxyribonuclease I Conjugates and Fluorescent phallotoxins (Molecular Probes, Eugene, OR), respectively, according to the manufacturer's instructions. Images were collected and analysed using Leica scanning confocal microscope (TCS SP2 AOB; Wetzlar, Germany).

### Xenograft assay

Animal studies were conducted according to protocols approved by Third Military Medical University Institutional Animal Care and Use Committee. Nude mice (5 weeks old) were purchased from Vital River Laboratories (VRL, Beijing, China), and inoculated subcutaneously with 2×10^6^ U937 cells into the lower back of each mouse. Mice were randomized into two groups (n=20). Five days after tumor inoculation, Mice were received AITC (50 mg/kg, i.p., five times per week) or an equal volume of vehicle. Tumor size and body weight were monitored per week after treatment, and the survival time of mice was recorded. Tumor tissues from representative mice were fixed in paraformaldehyde, embedded in paraffin, sectioned and processed for hematoxylin and eosin (H&E), terminal deoxynucleotidyl transferase-mediated dUTP nick end labeling assay (TUNEL) and immunohistochemical analysis.

### TUNEL, histological and immunohistochemical assay

TUNEL assay were performed by using an In Situ Cell Death Detection kit (Roche, Mannheim, Germany) according to the manufacturer’s instructions. Histological and immunohistochemical assay were performed as previously described [36].

### Statistical analysis

All of the data are expressed as mean ± SD of three individual experiments. Group measurements were compared using a Student’s *t*-test or analysis of variance (ANOVA). Survival analysis was performed with the Kaplan–Meier method, and significance was calculated using the log-rank test. *P < 0.05 or **P < 0.01 were considered significant.

## Abbreviations

ADF: Actin depolymerizing factor; AITC: Allyl isothiocyanate; AML: Acute myeloid leukemia; Apaf-1: Apoptotic protease activating factor-1; F-actin: Filamentous actin; G-actin: Globular actin; H&E: Hematoxylin and eosin; TUNEL: Erminal deoxynucleotidyl transferase-mediated dUTP nick end labeling.

## Competing interests

The authors declare that they have no competing interests.

## Authors’ contributions

NG and GL designed the experiments; GL, LL, TZ, CS, QC and JZ performed experiments; NG, GL, EL and PL analyzed data and wrote the paper. All authors read and approved the final manuscript.

## Supplementary Material

Additional file 1: Figure S1 AITC selectively induces apoptosis in a variety of leukemia cell lines through ROCK1/PTEN/PI3K-PP1/PP2A-cofilin pathway. **(A)** U937, Jurkat, and HL-60 cells were treated without or with 20 μM AITC for 24 h, Cell lysates, mitochondrial and cytosolic fractions were prepared and subjected to Western blot analysis. **(B)** Normal CD34+ cells were treated without or with 20 μM AITC for 24 h, Cell lysates, mitochondrial and cytosolic fractions were prepared and subjected to Western blot analysis. Click here for file
