# Supplementary figures and images for: Mitochondrial translocation of cofilin is required for allyl isothiocyanate-mediated cell death via ROCK1/PTEN/PI3K signaling pathway
Source: Cell Commun Signal. 2013 Jul 29;11:50. doi: 10.1186/1478-811X-11-50 (PMC3734051; doi:10.1186/1478-811X-11-50)

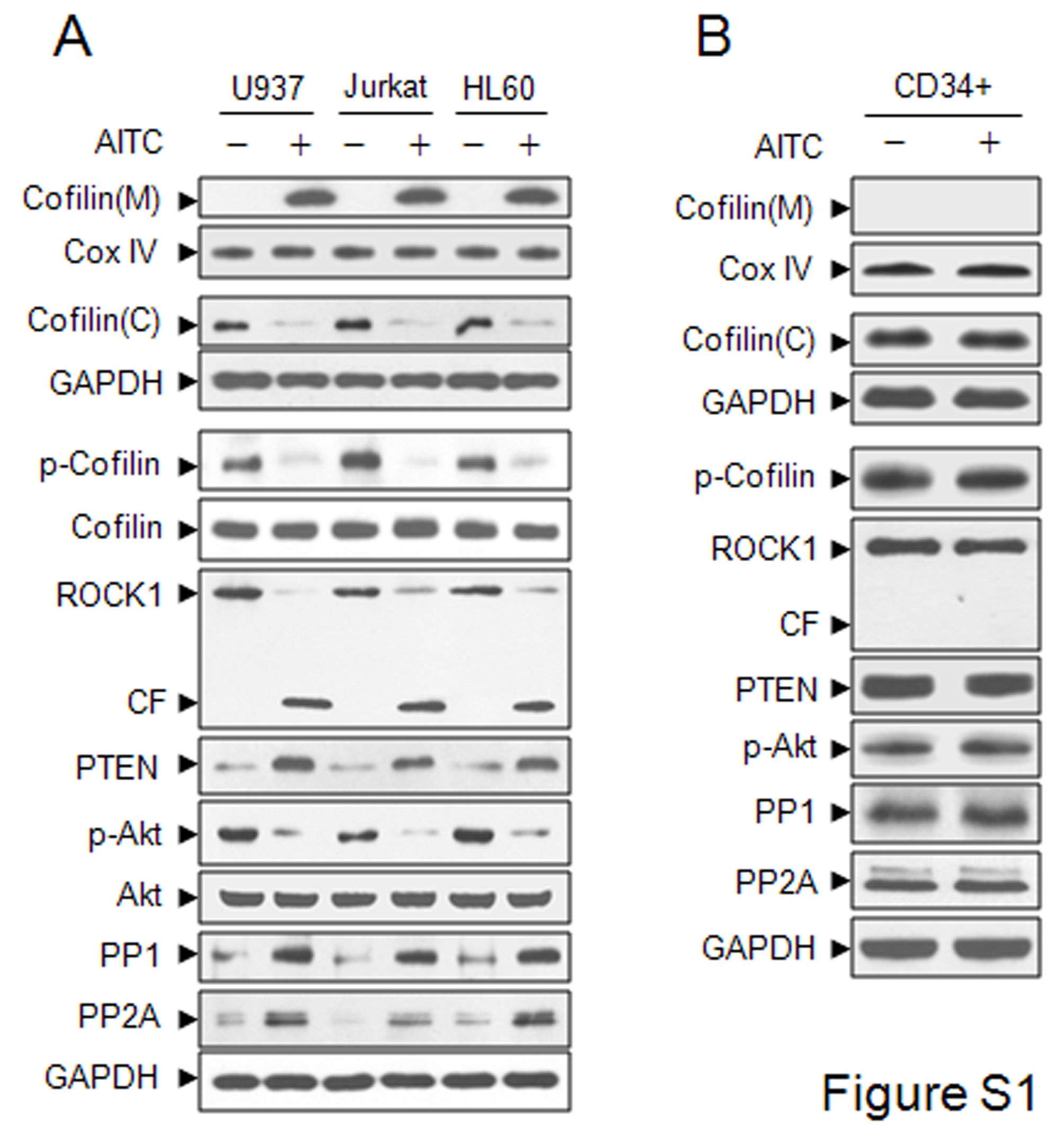

Supplement: Additional file 1: Figure S1 — AITC selectively induces apoptosis in a variety of leukemia cell lines through ROCK1/PTEN/PI3K-PP1/PP2A-cofilin pathway. (A) U937, Jurkat, and HL-60 cells were treated without or with 20 μM AITC for 24 h, Cell lysates, mitochondrial and cytosolic fractions were prepared and subjected to Western blot analysis. (B) Normal CD34+ cells were treated without or with 20 μM AITC for 24 h, Cell lysates, mitochondrial and cytosolic fractions were prepared and subjected to Western blot analysis. [file 1478-811X-11-50-S1.tiff]
